# Relationship between skin autofluorescence levels and clinical events in patients with heart failure undergoing cardiac rehabilitation

**DOI:** 10.1186/s12933-021-01398-0

**Published:** 2021-10-16

**Authors:** Mitsuhiro Kunimoto, Miho Yokoyama, Kazunori Shimada, Tomomi Matsubara, Tatsuro Aikawa, Shohei Ouchi, Kosuke Fukao, Tetsuro Miyazaki, Kei Fujiwara, Abidan Abulimiti, Akio Honzawa, Akie Shimada, Taira Yamamoto, Atsushi Amano, Masakazu Saitoh, Tomoyuki Morisawa, Tetsuya Takahashi, Hiroyuki Daida, Tohru Minamino

**Affiliations:** 1 Department of Cardiovascular Biology and Medicine, Juntendo University Graduate School of Medicine, 2-1-1 Hongo, Bunkyo-ku, Tokyo, 113-8421 Japan; 2 Cardiovascular Rehabilitation and Fitness, Juntendo University Hospital, Tokyo, Japan; 3 Department of Cardiovascular Surgery, Juntendo University Graduate School of Medicine, Tokyo, Japan; 4 Department of Physical Therapy Faculty of Health Science, Juntendo University, Tokyo, Japan

## Abstract

**Background:**

Advanced glycation end-products, indicated by skin autofluorescence (SAF) levels, could be prognostic predictors of all-cause and cardiovascular mortality in patients with diabetes mellitus (DM) and renal disease. However, the clinical usefulness of SAF levels in patients with heart failure (HF) who underwent cardiac rehabilitation (CR) remains unclear. This study aimed to investigate the associations between SAF and MACE risk in patients with HF who underwent CR.

**Methods:**

This study enrolled 204 consecutive patients with HF who had undergone CR at our university hospital between November 2015 and October 2017. Clinical characteristics and anthropometric data were collected at the beginning of CR. SAF levels were noninvasively measured with an autofluorescence reader. Major adverse cardiovascular event (MACE) was a composite of all-cause mortality and unplanned hospitalization for HF. Follow-up data concerning primary endpoints were collected until November 2017.

**Results:**

Patients’ mean age was 68.1 years, and 61% were male. Patients were divided into two groups according to the median SAF levels (High and Low SAF groups). Patients in the High SAF group were significantly older, had a higher prevalence of chronic kidney disease, and more frequently had history of coronary artery bypass surgery; however, there were no significant between-group differences in sex, prevalence of DM, left ventricular ejection fraction, and physical function. During a mean follow-up period of 590 days, 18 patients had all-cause mortality and 36 were hospitalized for HF. Kaplan–Meier analysis showed that patients in the high SAF group had a higher incidence of MACE (log-rank P < 0.05). After adjusting for confounding factors, Cox regression multivariate analysis revealed that SAF levels were independently associated with the incidence of MACE (odds ratio, 1.86; 95% confidence interval, 1.08–3.12; P = 0.03).

**Conclusion:**

SAF levels were significantly associated with the incidence of MACE in patients with HF and may be useful for risk stratification in patients with HF who underwent CR.

**Supplementary Information:**

The online version contains supplementary material available at 10.1186/s12933-021-01398-0.

## Introduction

Heart failure (HF) is closely associated with diabetes mellitus (DM), and patients with HF with DM have worse outcomes than those without DM [1]. The development of vascular complications associated with DM involves multiple dysfunction pathways, and among these damage pathways, advanced glycation end-products (AGEs) accumulation has gained particular attention [2]. Reducing sugars, such as glucose, are non-enzymatically bound to the amino groups of proteins, pass through Amadori compounds, and, after multiple reactions, change to irreversible AGEs [3]. AGEs rapidly accumulate in whole body tissues not only with aging but also under conditions such as hyperglycemia and chronic inflammation [4]. Moreover, AGEs form cross-links with vascular and muscle proteins, leading to physiological dysfunction in multiple organ systems [4]. AGEs that accumulate in the skin can be measured by a noninvasive method using the skin autofluorescence (SAF) value [5]. Recently, SAF level is reported to be useful as a prognostic marker for high-risk patients, such as patients with DM and chronic kidney disease (CKD) [6]. However, the relationship between SAF levels and clinical outcomes in patients with HF undergoing cardiac rehabilitation (CR) remains unclear. Thus, this study aimed to investigate the relationship between SAF levels and clinical prognosis in patients with chronic HF who have undergone CR.

## Methods

### Study population

**Fig. 1:**
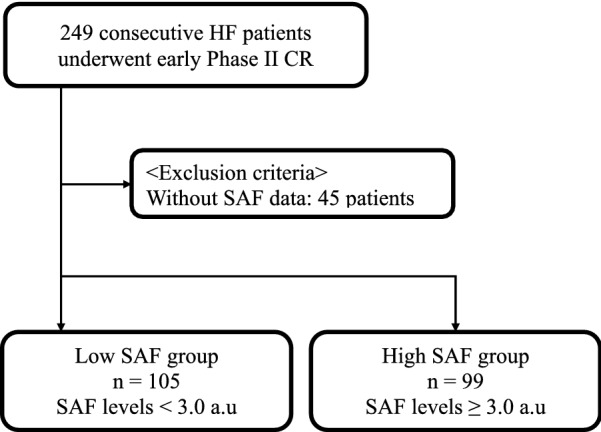
Study flowchart of all subjects. *HF* heart failure, *CR* cardiac rehabilitation, *SAF* skin autofluorescence

This was a retrospective observational study of 249 consecutive patients with HF who participated in phase II CR at our university hospital between November 2015 and September 2017. HF was defined according to the 2013 ACCF/AHA Guideline for the Management of HF [7]. Patients categorized as Stage B and C according to the ACCF/AHA classification that were determined to have indications for phase II CR by attending physician participated in the study. Stage D patients who could not undergo phase II CR were excluded. Of them, 45 patients who lacked baseline SAF data were excluded. The final study population consisted of 204 patients (Fig. 1). Written informed consent was provided by all patients before participation. The study protocol was approved by the ethics committee of our institution and conducted in accordance with the Declaration of Helsinki.

### Data collection and measurements

Age, sex, smoking history, comorbidities, and medical history were obtained from patient medical records. Blood samples were collected in the early morning after overnight fasting. A diagnosis of DM was defined by hemoglobin A1c level ≥ 6.5 or receiving treatment for DM. CKD was defined as an estimated glomerular filtration rate (eGFR) < 60 mL/min/1.73 m^2^, calculated using the renal disease equation with the Japanese coefficient using baseline serum creatinine level and modification to diet [8]. Body composition, grip strength, and SAF level were assessed at the beginning of CR. Anthropometric parameters, including body fat percentage, lean body weight, and muscle mass, were measured by bioelectrical impedance analysis (TANITA, MC-780A, Tokyo, Japan), as previously described [9]. Grip strength test was conducted in both hands in a standing position; the higher grip strength value was used. Exercise capacity was assessed using the cardiopulmonary exercise test (CPX) on a cycle ergometer (Strength ergo 8®) with an expiratory gas analysis machine (AE-310S®) using a ramp protocol to measure peak oxygen uptake (peakVO_2_) at the beginning of the CR.

### SAF

The SAF levels were measured by AGE Reader (DiagnOptics Technologies B.V., Groningen, The Netherlands) [5]. Briefly, the AGE Reader can noninvasively evaluate the accumulation of AGEs in the skin as the levels of fluorescence with excitation light [5]. SAF levels were calculated as the ratio of the average light intensity in the 420–600 nm wavelength range and average excitation light intensity in the 300–420 nm range. In the epithelium and dermis of the skin, AGEs are known to bind to collagen and elastin and accumulate [10]. A previous study on healthy subjects and those with DM showed that SAF levels assessed by the AGE Reader correlated well with the accumulation of AGEs, such as pentosidine and carboxymethyl-lysine, assessed by skin biopsy [11]. Therefore, SAF reflects mainly the accumulation of AGEs in the epithelium and dermis of the skin. Report has shown an intra-individual error percentage of approximately 5% for repeat skin AF measurements taken within a day [5]. Therefore, in the present study, SAF levels were measured only once from the inside of either forearm with patients in the sitting position.

### Primary endpoints

The primary outcome was major adverse cardiac events, defined as a composite of all-cause mortality and unplanned HF-related hospitalization. Mortality and hospitalization data were collected from the medical records of patients who died or were treated at our institution.

### Statistical analysis

Continuous variables were presented as mean ± standard deviation, and categorical variables were expressed as counts and percentages. Comparisons between groups were performed using Welch’s t-test for continuous variables and chi-squared test for categorical variables. Unadjusted cumulative event rate for the primary endpoint was estimated using the Kaplan–Meier method and compared between groups using the log-rank test. The cut-off value was defined using the median of the SAF level (3.0 AU). Univariate and multivariate Cox regression analyses were performed to identify the predictor of the primary endpoints. Hazard ratios and 95% confidence intervals (CIs) were also calculated. SAF, age, BMI, history of CABG, hemoglobin, albumin, eGFR, HbA1c, triglyceride, LDL-cholesterol, HDL-cholesterol, BNP, aspirin, statin, β-blockers, and oral hypoglycemic agent were selected as cofounding factors. Hazard ratios for continuous variables are expressed per SD change. Differences were considered significant at a P-value < 0.05. JMP version 12.0 (SAS Institute, Cary, NC, USA) was used to perform statistical analyses.

## Results

The mean patient age was 68 ± 15 years, and 125 patients (61.2%) were male. The patients were followed until December 2018. The mean follow-up duration was 590 ± 293 days*.*

Figure 1 presents the patient flowchart and exclusion criteria. Additional file 1: Figure S1 shows the distribution of SAF levels. The mean and median SAF levels were 3.0 ± 0.7 AU (mean ± SD) and 3.0 AU, respectively (range, 1.4–5.4 AU).

Based on the median SAF level (3.0 AU), patients were divided into two groups (High SAF and Low SAF). The High and Low SAF groups comprised 105 and 99 participants, respectively. Table 1 shows the clinical characteristics of the High and Low SAF groups. The two groups did not show significant difference in sex ratio. The High SAF group was significantly older and had a higher prevalence of CKD and history of coronary artery bypass graft. Hemoglobin, albumin, and eGFR levels were significantly lower, and fasting blood sugar and brain natriuretic peptide (BNP) levels were higher in the High SAF group. Cardiac function and grip strength were similar between the two groups. Patients in the High SAF group were more frequently prescribed with aspirin, statin, β-blockers, and oral hypoglycemic agent. CPX was performed in 55 and 33 cases in the Low and High SAF groups, respectively. The High SAF group had significantly lower exercise capacity compared to the Low SAF group. Additionally, we compared between DM and non-DM groups. Details are presented in Additional file 3: Table S2.

**Table 1:** Patient characteristics

	Low SAF (n = 99)	High SAF (n = 105)	P-value
Age	61.5 ± 16.0	74.3 ± 10.5	< 0.01
Male (%)	60 (60.6)	65 (61.9)	0.84
BMI	23.0 ± 4.0	23.1 ± 4.1	0.74
Hypertension (%)	50 (50.5)	60 (57.1)	0.34
Diabetes mellitus (%)	35 (35.4)	39 (37.1)	0.79
Dyslipidemia (%)	39 (39.4)	43 (41.0)	0.82
Chronic kidney disease (%)	45 (45.9)	65 (62.5)	0.02
Current smoking (%)	14 (14.3)	10 (9.6)	0.30
COPD (%)	1 (1.0)	5 (4.8)	0.11
History of MI (%)	12 (12.1)	22 (21.0)	0.08
History of PCI (%)	15 (15.2)	26 (24.8)	0.08
History of CABG (%)	6 (6.1)	24 (22.9)	< 0.01
History of valvular surgery (%)	10 (10.1)	13 (12.4)	0.60
History of CHF (%)	49 (49.5)	56 (53.9)	0.53
Valvular disease (%)			
Aortic valve stenosis	11 (11.1)	22 (21.0)	0.06
Aortic valve regurgitation	2 (2.0)	3 (2.9)	0.70
Mitral valve stenosis	1 (1.0)	1 (1.0)	0.96
Mitral valve regurgitation	15 (15.2)	27 (25.7)	0.06
Tricuspid valve regurgitation	15 (15.2)	13 (12.4)	0.57
Atrial fibrillation (%)	30 (30.3)	36 (34.3)	0.54
Dilated cardiomyopathy (%)	12 (12.1)	8 (7.6)	0.27
Echocardiography			
LVEF (%)	49 ± 20	51 ± 18	0.46
E/e′	19.1 ± 11.0	20.0 ± 11.7	0.77
Laboratory data			
Hemoglobin, g/dL	13.2 ± 2.3	12.2 ± 2.1	< 0.01
Albumin, g/dL	3.7 ± 0.5	3.6 ± 0.5	0.04
HbA1c (%)	6.0 ± 0.7	6.1 ± 0.9	0.36
Fasting blood sugar, mg/dL	97 ± 21	106 ± 30	0.02
Total cholesterol, mg/dL	167 ± 33	165 ± 38	0.69
LDL-cholesterol, mg/dL	98 ± 27	96 ± 32	0.76
HDL-cholesterol, mg/dL	46 ± 14	46 ± 12	0.85
Triglyceride, mg/dL	109 ± 54	104 ± 46	0.53
eGFR, mL/min/1.73 m^2^	66.5 ± 27.5	51.9 ± 25.0	< 0.01
BNP, pg/dL	325.4 ± 333.4	516.4 ± 862.7	0.04
Medication			
Aspirin (%)	27 (27.3)	52 (50.0)	< 0.01
ACE-I/ARB (%)	67 (67.7)	72 (69.2)	0.81
Statin (%)	44 (44.4)	63 (60.6)	0.02
β-Blocker (%)	70 (70.7)	87 (83.7)	0.03
Ca antagonist (%)	27 (27.3)	37 (35.6)	0.20
Loop diuretics (%)	63 (63.6)	75 (72.1)	0.19
Oral hypoglycemic agent (%)	10 (10.1)	22 (21.0)	0.03
Insulin (%)	4 (4.0)	9 (8.6)	0.19
Anthropometric data and grip strength			
Body fat percentage (%)	23.9 ± 7.8	24.9 ± 10.7	0.48
Lean body weight (kg)	46.7 ± 11.3	44.0 ± 9.9	0.11
Grip strength (kg)	28.0 ± 10.6	25.5 ± 8.1	0.23

### Endpoints

**Fig. 2:**
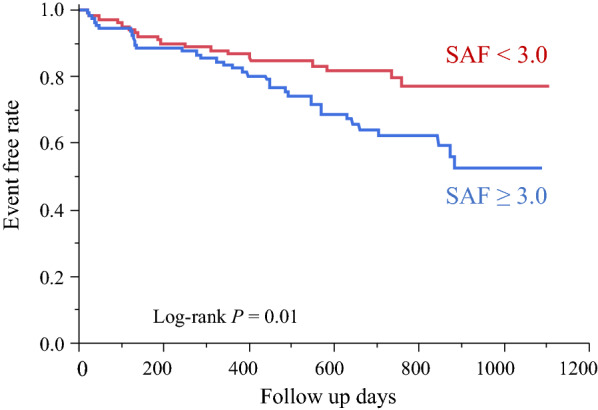
Kaplan–Meier survival curves. *SAF* skin autofluorescence

For the entire duration of follow-up, 18 patients in the Low SAF groups and 36 patients in the High SAF groups had a primary event (Additional file 2: Table S1). Kaplan–Meier analysis was performed to estimate the unadjusted event-free rate of primary endpoints. Event-free survival rate was significantly lower in the High SAF group than in the Low SAF group (P = 0.01, Fig. 2). Univariate Cox regression analyses revealed that SAF level was associated with the primary composite endpoint (odds ratio, 2.00; 95% CI 1.41–2.78; P < 0.01, Table 2). After adjustment for confounding variables, SAF level was significantly associated with long-term primary composite endpoint (odds ratio, 1.86; 95% CI 1.08–3.12; P = 0.03, Table 2).

**Table 2:** Univariate and multivariate Cox regression analyses of MACE

Variables	Univariate			Multivariate		
	Odds ratio	95% CI	P-value	Odds ratio	95% CI	P-value
SAF	2.00	1.41–2.78	< 0.01	1.86	1.08–3.12	0.03
Age	1.03	1.01–1.05	< 0.01	1.01	0.98–1.04	0.67
BMI	0.90	0.84–0.97	< 0.01	0.88	0.80–0.96	< 0.01
History of CABG	2.24	1.17–4.01	0.02	1.49	0.63–3.38	0.35
Hemoglobin	0.77	0.67–0.87	< 0.01	0.95	0.78–1.15	0.58
Albumin	0.54	0.30–0.96	0.03	1.93	0.86–4.30	0.10
eGFR	0.97	0.96–0.98	< 0.01	0.98	0.97–0.99	0.01
HbA1c	0.94	0.65–1.30	0.73	1.20	0.77–1.79	0.41
Triglyceride	0.99	0.99–1.00-	0.051	0.99	0.99–1.00	0.15
LDL-cholesterol	0.99	0.98–0.99	0.01	0.98	0.97–0.99	0.03
HDL-cholesterol	1.00	0.98–1.02	0.83	1.00	0.98–1.03	0.77
BNP	1.006	1.00–1.00	< 0.01	1.00	0.99–1.00	0.27
Aspirin	1.22	0.70–2.09	0.47	0.54	0.27–1.06	0.07
Statin,	1.18	0.69–2.05	0.55	1.24	0.57–2.69	0.59
β-Blockers	2.17	1.05–5.28	0.04	2.00	0.82–5.74	0.13
Oral hypoglycemic agent	1.09	0.50–2.11	0.83	0.84	0.30–2.10	0.72

*MACE* major adverse cardiovascular event, *CI* confidence interval, *SAF* skin autofluorescence, *BMI* body mass index, *CABG* coronary artery bypass graft, *eGFR* estimated glomerular filtration rate, *HbA1c* hemoglobin A1c, *LDL* low-density lipoprotein, *HDL* high-density lipoprotein, *BNP* brain natriuretic peptide

## Discussion

The present study demonstrated that higher SAF levels are significantly and independently associated with combined endpoint (all-cause death and unplanned HF-related hospitalization). To the best of our knowledge, this is the first study to demonstrate an association between SAF levels and adverse outcomes in patients with cardiovascular disease (CVD) who underwent CR.

In large clinical trials that analyze HbA1c levels, HbA1c was controlled at a lower level in the intensive care group than in the conventional treatment group, but macrovascular complications could not be significantly suppressed [12–14]. However, in the treatment of DM, strict glycemic control from the early stage of onset is extremely important. Previous studies have shown that, after long-term follow-up, adverse events, including death, were suppressed by the early intensive care group even after the intensive and conventional treatment groups eventually showed the same level of glycemic control [15, 16]. It is possible that early glycemic control may have long-term beneficial effects, so called “metabolic memory” [17]. AGEs may be used to explain the legacy effect because AGEs are metabolized very slowly and poorly degraded and remain in various types of diabetic tissue for a long period [4]. AGEs promote the production of intracellular oxidative stress after recognition by a receptor for AGEs (RAGE) and then activate nuclear factor κB and induce the secretion of multiple cytokines and growth factors and upregulation of adhesion factor [2]. The increase in oxidative stress caused by the AGE–RAGE system inactivates nitric oxide and promotes the inflammatory reaction and thrombotic tendency, leading to the onset and progression of arteriosclerosis [18, 19]. Accumulation of AGEs is associated with cardiovascular dysfunction [20]. In fact, the higher the concentration of AGEs in the blood, the higher the severity of HF, and AGEs are a poor prognostic factor in patients with HF [21, 22]. Moreover, AGEs have been associated with liver disease, lung disease, and malignant tumors [23–25].

Previous studies have reported that SAF levels were associated with vascular and multiorgan dysfunction [26] and cardiovascular adverse event in both high-risk patients and general populations [27–30]. Cavero-Redondo et al. reported that higher SAF levels were significantly associated with twofold risk of cardiovascular and all-cause mortality in high-risk patients [27]. In a study of patients with type 2 diabetes, Henderikus E Boersma reported that SAF was significantly associated with the development of new cardiovascular disease and death [31]. Waateringe et al. reported that, for every unit of SAF increase, the risk of developing CVD increases threefold and the risk of death increases fivefold in a study with 72,880 participants [30]. Additionally, a study on patients with HF with preserved ejection fraction found that higher SAF levels (SAF > 2.9 AU) were associated with first hospitalization for HF [32]. Moreover, our teams have recently reported that exercise capacity, a strong predictor in patients with CVD, was significantly lower in patients with higher SAF levels regardless of their DM status and that SAF levels were independently associated with exercise intolerance in patients with CVD [9]. The present study is the first to report that SAF levels are significantly associated with adverse outcomes in patients with HF who underwent CR. The clear cutoff value of SAF for diagnosis of risk remains unclear. The study population and number of events were relatively small; therefore, the cutoff value was defined using the median SAF level. However, even in the previous study [28], the median value was 2.69 AU (interquartile range, 2.26–3.19 AU), which is close to our data, and it seems to be appropriate. The present study revealed that eGFR levels were associated with primary endpoints. Previous studies had reported that lower eGFR was independently associated with death from any cause and HF hospitalization in patients with HF [33]. Moreover, other studies investigating SAF as a prognostic factor for cardiovascular events and death in patients with earlier stages of CKD revealed that both SAF and higher eGFR were independently associated with a decreased risk of cardiovascular events and death [34]. Our findings are consistent with those of previous reports. Univariate Cox regression analysis in the present study also found hemoglobin level to be associated with an increased risk of MACE. This vicious cycle of HF, renal failure, and anemia is known as cardiorenal anemia syndrome [35]. The relationship among the three factors causes vascular endothelial damage through overproduction of reactive oxygen species and impaired fluid regulation; furthermore, the progression of arteriosclerosis and increase in cardiovascular and renal load due to extracellular fluid retention are the main pathological conditions of this cycle [35]. We also identified BMI as a significant factor, with a 12% decrease in MACE for each 1-kg/m^2^ increase in BMI. Previous studies have reported that patients with HF who had higher BMI exhibited good prognosis, a phenomenon recognized as the “obesity paradox” [36]. Given that HF is a catabolic state, obese patients may exhibit better prognosis considering that they have more metabolic reserve [37, 38], allowing them to store more energy, which could be favorable for heart failure. Tumor necrosis factor (TNF)-α level has been known to increase in HF, thereby resulting in worsening of HF. Adipocytes produce antibodies that neutralize TNF-α, which acts as an anti-inflammatory [39]. Increased lean body mass was associated with better physical function, suggesting that skeletal muscle metabolism plays a central role in the exercise tolerance of patients with HF [40]. These facts may be associated with better clinical outcomes in such patients.

## Limitations

This study has several limitations. First, this study was conducted in a single center, and sample size was relatively small. Studies with a larger sample size will be more effective in evaluating the association between SAF levels and prognosis in patients with CVD who underwent CR. Second, we could not investigate other therapeutic interventions during the observation period. Third, we could not track the changes in SAF level over time. Fourth, the implementation rate of CPX was low. Fifth, SAF represents not only the fluorescence value generated by AGE of the skin but also that generated by other fluorophores, such as keratin [41]. Skin color and the use of skin creams can also affect SAF [42, 43]. However, only Japanese patients had been included herein, none of whom seemed to demonstrate excessive skin cream use. Although our data showed an association between SAF levels and adverse outcomes in patients with CVD, further intervention studies aimed at reducing AGE accumulation, as in previous studies [44], need to be conducted.

## Conclusion

SAF levels in patients with HF undergoing CR may be a predictor of all-cause mortality and HF hospitalization independent of multiple factors, and measurement of SAF level may provide useful information in patients undergoing CR.

## Supplementary Information

**Additional file 1: Figure S1.** Distribution of SAF levels.

**Additional file 2: Table S1.** Occurrence of composite endpoints.

**Additional file 3: Table S2.** Comparison of clinical characteristics between DM and non-DM groups.

## Data Availability

The datasets analyzed during the current study are not publicly available due to data protection regulations.

## Abbreviations

AGEs: Advanced glycation end-products
BMI: Body mass index
BNP: B-type natriuretic peptide
CI: Confidence interval
CKD: Chronic kidney disease
CR: Cardiac rehabilitation
CVD: Cardiovascular disease
DM: Diabetes mellitus
eGFR: Estimated glomerular filtration rate
HbA1c: Hemoglobin A1c
HDL: High density lipoprotein
HF: Heart failure
LDL: Low density lipoprotein
MACE: Major adverse cardiovascular event
MI: Myocardial infarction
SAF: Skin autofluorescence
TG: Triglyceride
