# Supplementary figures and images for: Relationship between skin autofluorescence levels and clinical events in patients with heart failure undergoing cardiac rehabilitation
Source: Cardiovasc Diabetol. 2021 Oct 16;20:208. doi: 10.1186/s12933-021-01398-0 (PMC8520614; doi:10.1186/s12933-021-01398-0)

Figure S1. Distribution of SAF levels

Number of patients (n)


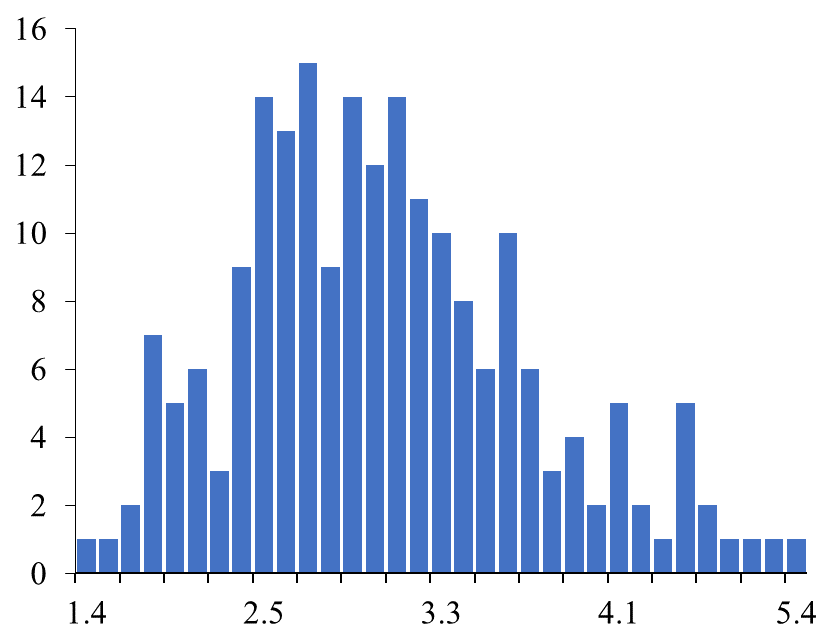


SAF, skin autofluorescence

Supplement: Supplementary file 1 — Additional file 1: Figure S1. Distribution of SAF levels. [file 12933_2021_1398_MOESM1_ESM.docx]
