# Supplementary material for: Relationship between skin autofluorescence levels and clinical events in patients with heart failure undergoing cardiac rehabilitation
Source: Cardiovasc Diabetol. 2021 Oct 16;20:208. doi: 10.1186/s12933-021-01398-0 (PMC8520614; doi:10.1186/s12933-021-01398-0)
Supplement: Supplementary file 2 — Additional file 2: Table S1. Occurrence of composite endpoints. [file 12933_2021_1398_MOESM2_ESM.docx]

Table S1. Occurrence of composite endpoints

|  | Low SAF group (n = 99) | High SAF group (n = 105) | P-value |
| --- | --- | --- | --- |
| Composite endpoints (%) | 18 (18.2%) | 36 (34.3) | <0.01 |
| All-cause mortality (%) | 6 (6.1) | 15 (14.3) | 0.05 |
| Heart failure | 2 | 3 |  |
| Cirrhosis | 2 | 0 |  |
| Cerebral stroke | 0 | 1 |  |
| Cancer | 0 | 1 |  |
| Pneumonia | 0 | 2 |  |
| Unknown | 2 | 8 |  |
| HF hospitalization (%) | 12 (12.1) | 21 (20.0) | 0.12 |

SAF, skin autofluorescence; HF, heart failure
