# Supplementary material for: Relationship between skin autofluorescence levels and clinical events in patients with heart failure undergoing cardiac rehabilitation
Source: Cardiovasc Diabetol. 2021 Oct 16;20:208. doi: 10.1186/s12933-021-01398-0 (PMC8520614; doi:10.1186/s12933-021-01398-0)
Supplement: Supplementary file 3 — Additional file 3: Table S2. Comparison of clinical characteristics between DM and non-DM groups. [file 12933_2021_1398_MOESM3_ESM.docx]

Table S2. Comparison of clinical characteristics between DM and non-DM groups.

|  | Non-DM (n = 130) | DM (n = 74) | P-alue |
| --- | --- | --- | --- |
| Skin autofluorescence (au) | 3.0±0.6 | 3.1±0.7 | 0.43 |
| Age | 67.5±15.9 | 69.1±13.0 | 0.48 |
| Male (%) | 74(56.9) | 51(68.9) | 0.08 |
| BMI | 22.1±3.7 | 24.8±4.0 | <0.01 |
| Hypertension (%) | 56(43.1) | 54(73.0) | <0.01 |
| Dyslipidemia (%) | 41(31.5) | 41(55.4) | <0.01 |
| Chronic kidney disease (%) | 59(45.7) | 51(69.9) | <0.01 |
| Current smoking (%) | 14(10.9) | 10(13.5) | 0.58 |
| COPD (%) | 5(3.9) | 1(1.3) | 0.28 |
| History of MI (%) | 16(12.3) | 18(24.3) | 0.03 |
| History of PCI (%) | 18(13.9) | 23(31.1) | <0.01 |
| History of CABG (%) | 11(8.5) | 19,25.68 | <0.01 |
| History of valvular surgery (%) | 14(10.8) | 9 (12.2) | 0.76 |
| History of CHF (%) | 58(44.6) | 47(64.4) | <0.01 |
| Valvular disease (%) | | | |
| Aortic valve stenosis | 21(16.2) | 12(16.2) | 0.99 |
| Aortic valve regurgitation | 4(3.1) | 1(1.3) | 0.42 |
| Mitral valve stenosis | 1(0.8) | 1(1.3) | 0.69 |
| Mitral valve regurgitation | 31(23.9) | 11(14.9) | 0.12 |
| Tricuspid valve regurgitation | 20(15.4) | 8(10.8) | 0.35 |
| Atrial fibrillation (%) | 35(26.9) | 31(41.9) | 0.03 |
| Dilated cardiomyopathy (%) | 12(9.2) | 8(10.8) | 0.71 |
| Echocardiography | | | |
| LVEF (%) | 52±19 | 47±18 | 0.058 |
| E/e' | 18.0±9.2 | 21.7±14.1 | 0.053 |
| Laboratory data | | | |
| Hemoglobin, g/dL | 12.5±2.3 | 12.9±2.1 | 0.21 |
| Albumin, g/dL | 3.7±0.5 | 3.7±0.5 | 0.49 |
| HbA1c (%) | 5.7±0.5 | 6.8±0.8 | <0.01 |
| Fasting blood sugar, mg/dL | 94±17 | 115±35 | <0.01 |
| Total cholesterol, mg/dL | 171±36 | 159±34 | 0.03 |
| LDL-cholesterol, mg/dL | 98±29 | 95±30 | 0.39 |
| HDL-cholesterol, mg/dL | 48±13 | 43±12 | 0.03 |
| Triglyceride, mg/dL | 100±49 | 117±49 | 0.02 |
| eGFR, mL/min/1.73 m^2^ | 63.4±29.2 | 51.1±21.3 | <0.01 |
| BNP, pg/dL | 425.2±767.9 | 408.1±393.6 | 0.86 |
| Medication | | | |
| Aspirin (%) | 44(33.9) | 35(48.0) | 0.04 |
| ACE-I/ARB (%) | 78(60.0) | 61(83.6) | <0.01 |
| Statin (%) | 59(45.4) | 48(65.8) | <0.01 |
| β-blocker (%) | 90(69.2) | 67(91.8) | <0.01 |
| Ca antagonist (%) | 38(29.2) | 26(35.6) | 0.34 |
| Loop diuretics (%) | 81(62.3) | 57(78.1) | 0.02 |
| α-glucosidase inhibitors | - | 7(9.4) | - |
| DPP-4 inhibitors | - | 28(37.8) | - |
| SGLT2 inhibitors | - | 5(6.8) | - |
| Glinides | - | 5(6.8) | - |
| Sulfonylureas | - | 7(9.5) | - |
| Metformin | - | 6(8.1) | - |
| Thiazolidinedione | - | 1(1.3) | - |
| GLP-1 | - | 1(1.3) | - |
| Insulin (%) | - | 15(20.2） | - |
| Anthropometric data and physical function | | | |
| Body fat percentage (%) | 23.3±9.2 | 26.2±9.3 | 0.054 |
| Lean body weight (kg) | 43.8±10.2 | 48.1±10.9 | 0.01 |
| Grip strength (kg) | 27.2±10.1 | 26.2±8.5 | 0.64 |
| Peak VO_2_ (mL/kg/min) | 15.8±3.8 | 15.1±4.3 | 0.47 |

Data are presented as mean ± standard deviation. BMI, body mass index; COPD, chronic obstructive pulmonary disease; MI, myocardial infarction; PCI, percutaneous coronary intervention; CABG, coronary artery bypass graft; CHF, congestive heart failure; CR, cardiac rehabilitation; LV, left ventricle; EF, ejection fraction; E, early diastolic filling velocity; A, late diastolic filling velocity; e’, early diastolic tissue velocity; eGFR, estimated glomerular filtration rate; TG, triglyceride; HDL, high-density lipoprotein cholesterol; LDL, low-density lipoprotein cholesterol; HbA1c, hemoglobin A1c; BNP, B-type natriuretic peptide; ACE-I, angiotensin converting enzyme inhibitor; ARB, angiotensin II receptor blocker; DPP-4, dipeptidyl peptidase-4; SGLT2, sodium-glucose transporter type 2; GLP-1, glucagon-like peptide-1; Peak VO_2_, peak oxygen uptake.
